# Generalization of Prigogine’s theorem for the case of full differential of entropy

**DOI:** 10.1016/j.mex.2018.11.009

**Published:** 2018-11-20

**Authors:** V.I. Shapovalov, N.V. Kazakov

**Affiliations:** a Moscow Humanitarian-Economical University, Volgograd Branch, Aviators avenue 8, Volgograd, Russia; b Volgograd Gazprom College, University prospect 71, Volgograd, Russia

**Keywords:** Application of the statistical criterion for the entropy change in an open system for analyzing the theorem on the minimum entropy production, irreversible processes, theorem on the minimum of entropy production, incomplete entropy differential, open system, entrostat

## Abstract

In this paper we propose a solution to the problem associated with the presence of an incomplete differential of entropy in Prigogine's theorem on the minimum of entropy production. A generalized theorem on the minimum rate of change of entropy near the stationary state of the system, which is under the influence of the entrostat, is formulated. It is shown that the mathematical expression of the generalized theorem contains the full differential of entropy.

• Our new method consists in applying of the statistical criterion for the entropy change in an open system (SCEC) for analyzing the I.
• Prigogine’s model in order to generalize the theorem on the minimum entropy production for the case of the total differential of entropy.

## Highlights

• We analyzed irreversible processes in a system under the influence of an entrostat
• We applied the statistical criterion for changing entropy for such a system
• We have changed the Prigogine’s formula for the case of the full entropy differential

## Graphical abstract

**Figure:**
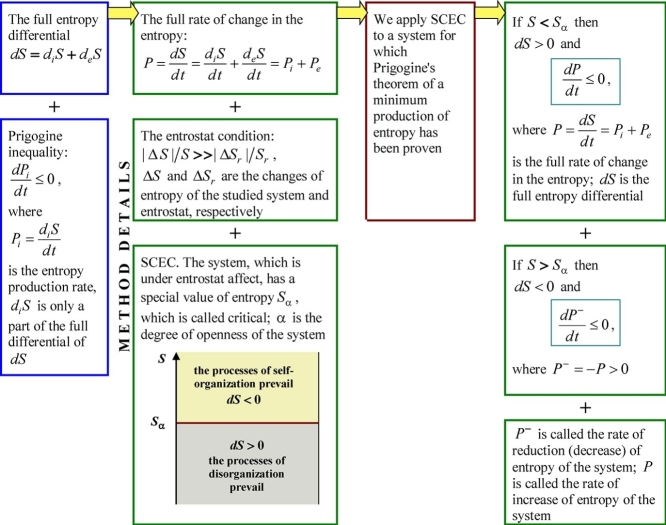

##  

**Specification Table**

**Table:** 

Subject Area	Physics and Astronomy
More specific subject area	Thermodynamics of nonequilibrium processes
Method name	Application of the statistical criterion for the entropy change in an open system for analyzing the theorem on the minimum entropy production.
Name and reference of original method	The original method is the statistical criterion for the entropy change in an open system (SCEC). It was first formulated by V.I. Shapovalov and is most fully described in the following works: 1) V.I. Shapovalov, Autom. Remote Control., 62 (2001), p. 909, 10.1023/A:1010245619531CrossRefView Record in Scopus 2) V.I. Shapovalov, Int. J. Appl. Math. Stat., 26 (2) (2012), p. 16, http://www.ceser.in/ceserp/index.php/ijamas/article/view/662View Record in Scopus 3) V.I. Shapovalov, N.V. Kazakov Nat. Sci., 6 (2014), p. 467, 10.4236/ns.2014.67045CrossRefView Record in Scopus
Resource availability	Our study relates to the theoretical field. Its results are based on the use of the concepts of statistical physics and the theory of nonequilibrium processes. Therefore, to reproduce our method, it is necessary to use the provisions of these theories and references from the previous paragraph.

## Introduction

In accordance with the law of increasing entropy, the entropy of a closed (isolated) system increases for irreversible processes: dS≥0. Here *S* is the entropy of the system and dS is the full entropy differential; an ‘=’ sign corresponds to the equilibrium state. For an open system the law of increasing entropy is not necessary – the sign of dS depends on the particular situation. In the middle of the last century, Prigogine proposed to split the total differential of an open system into two components [[1], [2], [3]]:

(1) $$dS=d_{i}S+d_{e}S.$$

Here, $d_{i}S$ is the non-negative entropy change produced inside the system, and $d_{e}S$ is the outflow or inflow of entropy into the system from the outside, with $d_{e}S$ sign depending on the situation. Theoretically, equation (1) can describe an open system in which entropy decreases, i.e., dS<0. For this, it is necessary that $d_{e}S<0$ and $\left|d_{e}S|\;>\;|d_{i}S\right|$. However, (1) does not contain indications of the factors on which the sign and quantity of $d_{e}S$ depend. For $d_{i}S$ whose sign is known, Prigogine proved the inequality:

(2) $$\frac{dP_{i}}{dt}\leq 0,$$

where *t* is time and $P_{i}=d_{i}S/dt$ is the entropy production rate in the system [3]. Note that *P_i_* contributes to the rate of change in the entropy, which we designated as *P*. The latter is expressed in terms of the total entropy differential of the system:

(3) $$P=\frac{dS}{dt}=\frac{d_{i}S}{dt}+\frac{d_{e}S}{dt}=P_{i}+P_{e}.$$

Analyzing (2), Prigogine formulated a theorem on the minimum production of entropy: in linear irreversible processes (under constant external conditions) the entropy production rate $P_{i}$ decreases and becomes minimal in the stationary state [1].

As this theorem specifies the direction of the process, it supplies presumably criterion of evolution for linear processes in an open system [4]. However, there are also some critics who consider that expression (2) does not allow for Prigogine's theorem to be a criterion of evolution (see in particular [5,6]). In their opinion, it is connected to the fact that, in (2), $P_{i}$ is expressed through $d_{i}S$ which is only a part of the full differential of dS [see (1)]. In other words, $P_{i}$ is expressed through incomplete differential of entropy. In comparison, the criterion of evolution of the closed system — the law of increase of entropy – contains full differential of dS.

It will be shown below that, for systems interacting with an entrostat, the inequality (2) can be replaced by another expression involving the total entropy differential.

## Method details

*Statistical criterion for entropy change*

Recall that the entrostat is the external system that does not change its entropy when it affects the system under investigation [7,8].

In practice the entrostat satisfies the following condition: $|\Delta S|/S>>\left|\Delta S_{r}\right|/S_{r}$, where ΔS and $\Delta S_{r}$ are the changes of entropy of the studied system and external system (caused by their interaction), respectively. For example, in physical systems the entrostat is a thermostat. Therefore, the role of the entrostat is played by an external system in which the change in entropy can be neglected in comparison with the change in entropy of the system under entrostat affect.

The main advantage of introducing the concept of an entrostat is that it allows for exclusion of the external environment when describing an open system. Indeed, since the parameters of the entrostat do not change when interacting with the system, all the variables are only related to the system itself. The latter fact made it possible to formulate and prove the so-called statistical criterion for entropy change (SCEC) in an open system [[7], [8], [9]]. Without enumerating all the provisions of this criterion, we confine ourselves to five that are relevant to the subject of this paper (see Additional information):

1. The system, which is under entrostat affect, has a special value of entropy, which is called critical.

2. If the entropy of the system is more than the critical value, then processes that decrease entropy (dS<0) prevail in the system; if the entropy is less than the critical value then processes that increase entropy (dS>0) predominate. When the critical value is reached, these processes balance each other, and the state of the system becomes stationary.

3. The critical value of the entropy of the system uniquely corresponds to the degree of openness of the system. In order to increase or decrease the critical value of entropy, it is necessary to reduce or increase the degree of openness of the system in relation to entrostat, respectively.

(Let us recall that degree of openness is understood to be the system parameter which quantitatively characterizes the value of impact of an entrostat on the system [[7], [8], [9]]. For example, in thermodynamics when describing the process of thermal conductivity in a plane-parallel plate located between two thermostats, the degree of openness of the system is the temperature difference between these thermostats.)

4. If openness degree increases, then the critical value of entropy decreases. According to the second point above, the entropy of the system has to decrease (dS<0) to the new value corresponding to a new degree of openness.

5. If openness degree decreases, then the critical value of entropy increases. According to the second point above, the entropy of the system has to increase (dS>0) to the new value corresponding to a new degree of openness.

*Generalized theorem on the minimum rate of change of entropy near the stationary state of the system*

Let us apply this criterion to a system [1] for which Prigogine's theorem of a minimum production of entropy has been proven.

Using the conception of Local Equilibrium, we introduce the notation of known values:

s=dS/dV – local entropy, i.e. entropy per unit volume (recall that $s=\rho \hat{s}$, where ρ is the density; $\hat{s}=dS/\left(\rho dV\right)$ is the specific local entropy, i.e. the entropy per unit mass); *V* is volume and *S* is the entropy of the system;

σ = ds/dt  – rate of change of local entropy (according to (1) $\sigma =\sigma_{i}+\sigma_{e}$ where $\sigma_{i}$ – the local production of entropy);

P=dS/dt=∫σ  dV – rate of change in the entropy of the system.

Pay attention; in all of these expressions dS is the full differential of entropy.

Proceeding as in [1], we assume that the linear components of the processes prevail near the stationary state. This assumption allows us to apply the following known equations: $\sigma =\sum X_{n}I_{n}$, $I_{n}=\sum L_{nk}X_{k}$ and $L_{nk}=L_{kn}$, where $X_{n}\;$ is a generalized force, $I_{n}\;$ is a generalized flow, $L_{nk}$ are kinetic coefficients. Our calculations differ from that ones [1] by the fact that we use the rate of change of local entropy σ instead of local entropy production.

Consider a system consisting of two vessels separated by a partition. These vessels have different temperatures and composition of the substances inside them. A constant temperature difference is maintained between the vessels. The partition is removed and, at the same time, two streams appear in the system: the flow $I_{1}$ arising due to temperature differences, and flow $I_{2}$ arising due to the unequal composition of the vessels. As a result, after some time the system goes into a stationary state. The flow $I_{1}$ corresponds to the generalized force $X_{1}$. The diffusive flow $I_{2}$ corresponds to the generalized force $X_{2}$.

Let us remind that we consider the rate of local change of entropy σ instead of local production of entropy $\sigma_{i}$. It is obvious that $\sigma {|}_{st}=0$ (“*st*” means “stationary state”). Then it follows from the equation $\sigma =\sum X_{n}I_{n}$ that $X_{1}{|}_{st}=0$ i.e. after the disappearance of the septum, this force gradually decreases to zero. Consequently, $X_{1}$ is a variable (like $X_{2}$ force), which slightly differs our line of reasoning from [1]. In the absence of $X_{1}$ force, the flow $I_{1}$ disappears, i.e. $I_{1}{|}_{st}=0$. Also $I_{2}{|}_{st}=0$, since diffusion stops when the system reaches a stationary state.

As a result, we come to the ratios similar to those found in [1] except that they contain σ instead of $\sigma_{i}$ and both generalized forces are variable:

(4) $$\begin{array}{l} \sigma =I_{1}X_{1}+I_{2}X_{2}\\ I_{1}=L_{11}X_{1}+L_{12}X_{2}\;,\;I_{2}=L_{21}X_{1}+L_{22}X_{2}\\ \sigma =L_{11}X_{1}^{2}+2L_{21}X_{1}X_{2}+L_{22}X_{2}^{2}=L_{11}X_{1}^{2}+2L_{12}X_{1}X_{2}+L_{22}X_{2}^{2}\;. \end{array}$$

As can be seen from (4), σ is a function of two variables: $X_{1}$ and $X_{2}$. To determine its extremum, we find the corresponding derivatives:

(4a) $$\begin{array}{c} \frac{\partial \sigma }{\partial X_{2}}=2(L_{21}X_{1}+L_{22}X_{2})=2I_{2}\;;\\ \frac{\partial \sigma }{\partial X_{1}}=2(L_{11}X_{1}+L_{12}X_{2})=2I_{1}\;; \end{array}$$

(5) $$\frac{\partial^{2}\sigma }{\partial X_{2}^{2}}=2L_{22}=A\;;$$

$\frac{\partial^{2}\sigma }{\partial X_{2}\partial X_{1}}=2L_{21}=B$ and $\frac{\partial^{2}\sigma }{\partial X_{1}^{2}}=2L_{11}=C$.

Since $I_{2}{|}_{st}=0$ and $I_{1}{|}_{st}=0$ (see above), then in (4a) both derivatives are equal to zero in the stationary state.

We got that in linear processes in a stationary state, the rate of change of the local entropy σ, and consequently the entropy change rate *P*, can have an extremum.

To determine the existence and type of extremum, it is necessary to find the sign of discriminant $D=AC-B^{2}=4(L_{22}L_{11}-L_{21}^{2})$ and sign $A=2L_{22}$ (or sign $C=2L_{11}$).

For this purpose we take into account that in Prigogine’s model the thermostat maintains a constant temperature difference, i.e. it is an entrostat. Therefore, we can apply SCEC.

Let us consider two cases (only for systems under the influence of the entrostat).

Case 1 Suppose that the degree of openness of the system has increased, for example, the temperature difference between the vessels has increased. According to the fourth point of SCEC, the system will tend towards a state with lower entropy. Hence, σ<0 and P<0. Let us make a replacement of variables: $\sigma^{-}=-\sigma \;>0$ and $P^{-}=-P>0$. It is easy to see that previous arguments remain valid for the new variables. Really,

$$\sigma^{-}=-\frac{ds}{dt}=\frac{d(-s)}{dt}=\sum X_{n}I_{n}\;,$$

where $X_{n}\;=\;\partial (-s)/\partial a_{n}\;$; $a_{n}$ – local thermodynamic parameter. As a result we come to a positive square form for $X_{n}$ :

$$\sigma^{-}=L_{11}X_{1}^{2}+2L_{21}X_{1}X_{2}+L_{22}X_{2}^{2}\;>0$$

[see (4)]. We will find eigenvalues for the matrix of coefficients of this square form:

$$\lambda_{1}=\frac{1}{2}\left(L_{11}+L_{22}+\sqrt{{\left(L_{11}-L_{22}\right)}^{2}+4L_{12}^{2}}\right)\;\;>0\;;$$

$$\lambda_{2}=\frac{1}{2}\left(L_{11}+L_{22}-\sqrt{{\left(L_{11}-L_{22}\right)}^{2}+4L_{12}^{2}}\right)\;\;>0\;.$$

Here, we have taken into account that the signs of the eigenvalues coincide with the sign of the quadratic form. We find from the second inequality that $L_{11}L_{22}\;>\;L_{12}^{2}$. From where it follows that a) both coefficients have the same signs and b) D>0, *i.e.* $\sigma^{-}$ has an extremum in a stationary state. Adding the inequalities we obtain $L_{11}+L_{22}>0$. Therefore, both coefficients are positive and A>0.

Thus, in the stationary state the extremum of the functions $\sigma^{-}$ and $P^{-}$ is a minimum. Mathematically, this statement can be written as

(6) $$\frac{dP^{-}}{dt}\leq 0$$

(‘=’ corresponds to a stationary state).

We will call value of $P^{-}$ the rate of reduction (decrease) of entropy of the system.

It follows from (6) that in linear processes, as the system approaches the stationary state, this rate should decrease.

Case 2 Let us assume that the degree of openness of the system has decreased, for example, we have reduced the difference in temperature between the vessels. According to the fourth SCEC point, the system will tend to a state with more entropy: σ>0 and P>0. The quadratic form in the expression for σ is positive. It means that we come to the inequality

(7) $$\frac{dP}{dt}\leq 0$$

(‘=’ corresponds to a stationary state).

Inequalities (6) and (7) are a mathematical expression of the generalized theorem on the minimum rate of change of entropy, of an open system under the influence of an entrostat. Its formulation states that, in linear processes (with a constant value of the entrostat influence), as the system approaches the stationary state, *the rate of change* of its entropy decreases and reaches a minimum in the stationary state [9]. If processes that decrease entropy predominate in the system, then the rate of *decrease of entropy* decreases. If the processes that increase entropy prevail in the system, the rate of *increase of entropy* decreases.

In conclusion this Section, we note that the mathematical form of the inequalities (6) and (7) allows for changes in the sign of the second (at least) order time derivative of entropy. This indicates the theoretical possibility of the appearance of entropic oscillations in a neighborhood of the stationary state [9]. In our opinion, this is an essential step in the theory of entropy processes. In particular, the well-known inequality (2) prohibits entropy oscillations. Indeed, in the oscillation equation, the second (as a minimum) order derivative of the entropy should periodically change sign: $d^{2}S/dt^{2}$. However, it follows from (2) that this derivative always has only one sign, which is negative: $dP_{i}/dt=d_{i}^{2}S/dt^{2}\leq 0$. In our opinion, this restriction is due to the fact that, in (2) $P_{i}$ manifests through an incomplete differential of entropy. Thanks to (6) and (7), this specified restriction disappears for systems under the influence of the entrostat.

## Conclusions

1 We have shown in this paper that inequalities (6) and (7) replace formula (2) for a system under the influence of entrostat. Their advantage is that they are written not for the entropy production rate $P_{i}$, as in formula (2), but for the full rate of change of the entropy *P*, described in (3). We emphasize that *P* is expressed in terms of the full entropy differential dS, but $P_{i}$ is expressed in terms of an incomplete differential of entropy. Therefore, (6) and (7) have a higher level of generality than (2), and therefore can apply the evolution criterion for linear processes.
2 What the novelty of our approach consists in? We have found out that thermostat is an entrostat in the task considered by Prigogine. The presence of entrostat allowed us to apply a new method, including SCEC. Further we showed that if we apply SCEC, then the arguments of Prigogine himself will be quite enough to reformulate the theorem on the minimum of entropy production for the case of a full entropy differential.

## Additional information

*Entrostat and SCEC concepts*

The concept of the entrostat appeared in order to resolve the crisis in statistical physics that called into question the interpretation of entropy as a measure of disorder. The main argument of the opponents of this interpretation was the fact that statistical physics failed to provide an explanation of the phenomenon of self-organization [8].

The main obstacle to the explanation of self-organization in statistical theory is the difficulty comparing the entropies of different states of an open system. What do we mean by this? If the system under study interacts with another system, changes occur in both of them. To describe these changes, the appropriate variables are required. If these two systems constitute a general closed system, then in principle we can determine all the changes in each of them. Herewith, in the statistical expression of entropy (Boltzmann-Gibbs entropy) written for the system under study, the distribution function (the probability density) becomes conventional, depending on the variables of both systems. However, in most real cases, the system under study is affected by a very large number of external systems – the external environment. In such cases, it is almost impossible to consider all the required variables and, therefore, it is impossible to record properly the statistical expression of entropy. The introduction of the concept of the entrostat allowed researchers to address the issue of comparing the entropies of different states from an unexpected viewpoint. As a result, the study of self-organization within statistical theory made headway [8].

The main advantage of introducing the concept of the entrostat is that it excludes the external environment when studying the behavior of an open system. In particular, all the changes that occur during the interaction of the system with the entrostat relate to the system. Therefore, all the new variables that describe these changes will relate to the system. The latter means that we do not need to build a general closed system.

For a system under the influence of an entrostat, the following inequality was proved [[7], [8], [9]]

(8) $$S[X]\;>\;S[X|Y_{1}]\;>\;S[X|Y_{1}Y_{2}]\;>\;\ldots \;>\;S[X|Y_{1}Y_{2}\ldots Y_{i}]\;>\;\ldots \;>0\;,$$

where square brackets indicate accessory and do not mean functional dependence; vertical line means ‘under a condition’. In this formula:

S[X]=∫f(X)lnf(X)dX

– entropy of the closed system in an equilibrium state (here we use the designations entered in [10]: f(X)=dp/dX – distribution function of a set of random variables *X*; *p* – probability; *X* – the point of the phase space in which the normality condition is satisfied ∫f(X)dX=1);

$$S\left[X|Y_{1}\right]=\iint f\left(XY_{1}\right)\ln f\left(X|Y_{1}\right)dXdY_{1}$$

– the entropy of the system in a stationary state, in which the action of an entrostat creates a change in the system described by the variable $Y_{1}$;

$$S\left[X|Y_{1}Y_{2}\ldots Y_{i}\right]=\int \ldots \int f\left(XY_{1}Y_{2}\ldots Y_{i}\right)\ln f\left(X|Y_{1}Y_{2}\ldots Y_{i}\right)dXdY_{1}dY_{2}\ldots dY_{i}$$

– the entropy of the system in a stationary state in which the action of an entrostat creates the changes in system described by variables $Y_{1}\text{,}Y_{2},\ldots ,Y_{i}$.

To prove inequality (8) let us compare two stationary states, differing in the magnitude of the effect of the entrostat on the system [7,9]. The first state differs from the closed state by the changes described by the variables *Y*_1_*,Y*_2_*,…,Y_n-_*_1_. The second state differs by the changes described by the variables *Y*_1_*,Y*_2_*,…,Y_n-_*_1_*,Y_n_*. As everybody can see, the system is under greater external effect in the second state in comparison with the first one. We wrote the expressions for the corresponding conditional entropies:

(9) $$S\left[X|Y_{1}...Y_{n-1}\right]=-k\int_{X}\int_{Y_{1}}...\int_{Y_{n-1}}f\left(XY_{1}...Y_{n-1}\right)\ln f\left(X|Y_{1}...Y_{n-1}\right)dX...dY_{n-1}$$

– in the first state;

(10) $$S\left[X|Y_{1}...Y_{n}\right]=-k\int_{X}\int_{Y_{1}}...\int_{Y_{n}}f\left(XY_{1}...Y_{n}\right)\ln f\left(X|Y_{1}...Y_{n}\right)dX...dY_{n}$$

– in the second state.

In these formulas $f\;(X|Y_{1}...Y_{n-1})$ and $f\;(X|Y_{1}...Y_{n})$ are one-dimensional conditional density distribution of probability, $f\;(XY_{1}...Y_{n-1})$ and $f\;(XY_{1}...Y_{n})\;$ are multidimensional density distribution of probability. Let us write some known relations between multidimensional distributions and one-dimensional conditional distributions, which we will apply in the following:

(11) $$\begin{array}{l} f(XY)=f(X|Y)f(Y)=f(Y|X)f(X)\;;\\ f(XY_{1}Y_{2})=f(X|Y_{1}Y_{2})f(Y_{1}Y_{2})=f(Y_{1}|XY_{2})f(XY_{2})=f(Y_{2}|XY_{1})f(XY_{1})\;;\\ f(XY_{1}...Y_{n})=f(X|Y_{1}...Y_{n})f(Y_{1}...Y_{n})=f(Y_{1}|XY_{2}...Y_{n})f(XY_{2}...Y_{n})=...=f(Y_{n}|XY_{1}...Y_{n-1})f(XY_{1}...Y_{n-1})\;. \end{array}$$

We show that (9) can be represented as [7,9]

(12) $$S\left[X|Y_{1}...Y_{n-1}\right]=-k\int_{X}\int_{Y_{1}}...\int_{Y_{n}}f\left(XY_{1}...Y_{n}\right)\ln f\left(X|Y_{1}...Y_{n-1}\right)dX...dY_{n}\;.$$

For this, we transform the integral in (12) to the form of the integral in (9):

$$\underset{X}{\int }\int_{Y_{1}}...\int_{Y_{n}}\underset{see\;\left(11\right)}{\underbrace{f\left(XY_{1}...Y_{n}\right)}}\ln f\left(X|Y_{1}...Y_{n-1}\right)dX\ldots dY_{n}=\int_{X}\int_{Y_{1}}...\int_{Y_{n}}f\left(Y_{n}|XY_{1}...Y_{n-1}\right)f\left(XY_{1}...Y_{n-1}\right)\ln f\left(X|Y_{1}...Y_{n-1}\right)dX\ldots dY_{n}=$$

$$=\underset{X}{\int }\int_{Y_{1}}...\int_{Y_{n-1}}f\left(XY_{1}...Y_{n-1}\right)\ln f\left(X|Y_{1}...Y_{n-1}\right)dX\ldots dY_{n-1}\int_{Y_{n}}\underset{=1}{\underbrace{f\left(Y_{n}|XY_{1}...Y_{n-1}\right)}}dY_{n}=\int_{X}\int_{Y_{1}}...\int_{Y_{n-1}}f\left(XY_{1}...Y_{n-1}\right)lnf\left(X|Y_{1}...Y_{n-1}\right)dX\ldots dY_{n-1}\;.$$

Thus, (12) is proved.

Let us prove the inequality

(13) $$S\left[X|Y_{1}...Y_{n-1}\right]\;>\;S\left[X|Y_{1}...Y_{n}\right]:$$

For this purpose, using (10), (12) and (11), we will find the sign of a difference $S\left[X|Y_{1}...Y_{n-1}\right]-S\left[X|Y_{1}...Y_{n}\right]$:

$$S\left[X|Y_{1}...Y_{n-1}\right]-S\left[X|Y_{1}...Y_{n}\right]=k\int_{X}\int_{Y_{1}}...\int_{Y_{n}}f\left(XY_{1}...Y_{n}\right)\ln\frac{f\left(X|Y_{1}...Y_{n}\right)}{f\left(X|Y_{1}...Y_{n-1}\right)}dX...dY_{n}\;>$$

$$>\;k\underset{X}{\int }\int_{Y_{1}}\ldots \int_{Y_{n}}f\left(XY_{1}...Y_{n}\right)\left[1-\frac{f\left(X|Y_{1}...Y_{n-1}\right)}{f\left(X|Y_{1}...Y_{n}\right)}\right]dX\ldots dY_{n}=\underset{=1}{k\underbrace{\underset{X}{\int }\int_{Y_{1}}\ldots \int_{Y_{n}}f\left(XY_{1}...Y_{n}\right)dX\ldots dY_{n}}}-k\underset{X}{\int }\int_{Y_{1}}\ldots \int_{Y_{n}}f\left(XY_{1}...Y_{n}\right)\frac{f\left(X|Y_{1}...Y_{n-1}\right)}{f\left(X|Y_{1}...Y_{n}\right)}dX\ldots dY_{n}=$$

$$=k-k\underset{X}{\int }\int_{Y_{1}}\ldots \int_{Y_{n}}f\left(Y_{1}...Y_{n}\right)f\left(X|Y_{1}...Y_{n-1}\right)dX\ldots dY_{n}=k-k\int_{Y_{1}}\ldots \int_{Y_{n}}f\left(Y_{1}...Y_{n}\right)\underset{=1}{\underbrace{\int_{X}f\left(X|Y_{1}...Y_{n-1}\right)dX}}dY_{1}\ldots dY_{n}=k-k=0\;.$$

The well-known proportion were used in this transformation:

lna>1−1/a;a≠1.

Thus, the inequality (13) is proved. The number n is arbitrary, hence the inequality (13) can be written down in an expanded form (8) [7,9].

The entropies in the inequality (8) correspond to stationary states that differ in the magnitude of the entrostat effect. The greater the magnitude of this impact is, - much more changes there are in the system. In inequality (8), this proposal is reflected in the increase of the variables number in each subsequent inequality. Let us denote as degree of openness of a system is a parameter that characterizes the number of variables needed to describe the changes that appear in the system under the influence of the entrostat. We denote this parameter by $\alpha_{i}$. In particular, for a closed system $\alpha_{i}=0$. As can be seen from (8), there is the unique compliance between degree of openness of system $\alpha_{i}$ and the stationary entropy value $S[X|Y_{1}Y_{2}\ldots Y_{i}]$:

$$S\left[X|Y_{1}Y_{2}\ldots Y_{i}\right]\Leftrightarrow \alpha_{i}$$

To make the further presentation clearer, we introduce the designation: $S[X|Y_{1}Y_{2}\ldots Y_{i}]=S_{\alpha i}$. Then, (8) can be represented as:

(14) $$\begin{array}{l} S_{\alpha =0}\;>\;S_{\alpha 1}\;>\;S_{\alpha 2}\;>\;\ldots \;>\;S_{\alpha i}\;>\;\ldots \;>\;0\;,\\ \;\;0\;\;<\;\;\;\alpha_{1}\;<\;\;\;\alpha_{2}\;<\;\;\;\ldots \;<\;\;\;\alpha_{i}\;<\ldots \;<\;\alpha_{\max}\;. \end{array}$$

The stationary entropy value $S_{\alpha i}$ was called critical. The introduction of this term is caused by the following reasons. First, in irreversible processes only in a stationary state the entropy of system does not change ($S_{\alpha i}$ = *const*). Secondly, the stationary entropy value $S_{\alpha i}$ uniquely corresponds to openness degree $\alpha_{i}$. Thirdly, let the degree of openness be constant in a certain period of time, then a) if the entropy of the system is less than the critical value ($S\;<\;S_{\alpha i}$), then the processes that increase entropy to $S_{\alpha i}$ prevail in the system, after which the entropy ceases to change; b) if the entropy of the system is greater than the critical value ($S\;>S_{\alpha i}$), then the processes that reduce the entropy to $S_{\alpha i}$ prevail in the system, after which the entropy ceases to change. It follows from this that the stationary value $S_{\alpha i}$ separates two opposite processes in the system. Therefore it was called critical.

These arguments were used as the basis for the formulation of the SCEC.

In conclusion of this Section, we make an important remark [8,9]: if the system interacts with another system that is not an entrostat, then (8) and (14) are not satisfied. For such a system SCEC can not be used. In particular, (8) does not hold in the well-known thermodynamic experience: two systems with different temperatures are in thermal contact and together form a heat-insulated common system; the entropy of one system surely decreases, and the entropy of the other necessarily increases. The latter, as it would seem, contradicts (8), since according to this inequality the entropy of each system should decrease. However, none of the two described systems is an entrostat, and therefore inequality (8) can not be applied in this case.

*Some issues for discussion*

We used the concept of entrostat and applied SCEC in a model that has been carefully studied by many researchers. However, these concepts are completely absent in publications on this theme. This fact makes our work and our method rather unusual. As a result, discussion questions must inevitably arise. In this section, we would like to discuss some of those ones that were raised during the manuscript preparation for publication.

1. In our work, we expressed the rate of change of the local entropy of the system σ=ds/dt (wheres=dS/dV, where *S* is the entropy of the system; *V* is the volume) through the multiplication of generalized forces *X* and flows *I*. This fact caused serious criticism of some of our colleagues. In their opinion, this multiplication is valid only for the local production of entropy $\sigma_{i}$ (the notation adopted in our article) and cannot be applied to σ. Below we give arguments in favor of our position.

1.1 Mathematical model

$$s=\frac{dS}{dV}=\frac{d_{e}S+d_{i}S}{dV}=\underset{s_{e}}{\underset{︸}{\frac{d_{e}S}{dV}}}+\underset{s_{i}}{\underset{︸}{\frac{d_{i}S}{dV}}}=s_{e}+s_{i}.$$

where the index “*e*” means “external”, the index “*i*” means “internal”.

(15) $$\sigma =\frac{ds}{dt}=\frac{ds_{e}+ds_{i}}{dt}=\underset{\sigma_{e}}{\underbrace{\frac{ds_{e}}{dt}}}+\underset{\sigma_{i}}{\underbrace{\frac{ds_{i}}{dt}}}=\sigma_{e}+\sigma_{i}\;,$$

(16) $$\left\{\begin{array}{c} s_{i}=s_{i}(a_{1},a_{2},\ldots ,a_{n});\;s_{e}=s_{e}(b_{1},b_{2},\ldots ,b_{m});\\ s=s(a_{1},a_{2},\ldots ,a_{n},b_{1},b_{2},\ldots ,b_{m})=s(c_{1},c_{2},\ldots ,c_{k}). \end{array}\right)$$

(17) $$\sigma =\frac{ds}{dt}=\sum_{k}\frac{\partial s}{\partial c_{k}}\frac{dc_{k}}{dt}=\sum_{k}\left(\frac{\partial s_{e}}{\partial c_{k}}+\frac{\partial s_{i}}{\partial c_{k}}\right)\frac{dc_{k}}{dt}=\sum_{k}X_{k}^{\left(\sigma \right)}I_{k}^{\left(\sigma \right)}\;.$$

$$\sigma_{i}=\frac{ds_{i}}{dt}=\sum_{n}\frac{\partial s_{i}}{\partial a_{n}}\frac{da_{n}}{dt}=\sum_{n}X_{n}^{\left(i\right)}I_{n}^{\left(i\right)}\;.$$

(18) $$\sigma_{e}=\frac{ds_{e}}{dt}=\sum_{m}\frac{\partial s_{e}}{\partial b_{m}}\frac{db_{m}}{dt}=\sum_{m}X_{m}^{\left(e\right)}I_{m}^{\left(e\right)}\;.$$

$X_{k}^{\left(\sigma \right)}=\frac{\partial s_{e}}{\partial c_{k}}+\frac{\partial s_{i}}{\partial c_{k}}$ (see (17)).

In accordance with (16), all ${\partial s}_{e}/\partial \left(c_{k}-b_{m}\right)$and ${\partial s}_{i}/\partial \left(c_{k}-a_{n}\right)$ disappear, therefore $\frac{\partial s_{e}}{\partial c_{k}}=\frac{\partial s_{e}}{\partial b_{m}}$ and $\frac{\partial s_{i}}{\partial c_{k}}=\frac{\partial s_{i}}{\partial a_{n}}$,

Consequently, $X_{k}^{\left(\sigma \right)}=X_{m}^{\left(e\right)}+X_{n}^{\left(i\right)}$. Note that in the general case $I_{k}^{\left(\sigma \right)}\;\neq \;I_{m}^{\left(e\right)}\;\neq \;I_{n}^{\left(i\right)}$, since $dc_{k}/dt\neq db_{m}/dt\neq da_{n}/dt$.

1.2. Using the example of a simple task, we will demonstrate that our approach does not contradict the known provisions of nonequilibrium thermodynamics

Consider a homogeneous system with a temperature gradient in one direction *x*.

We write the entropy balance equation in general form:

(19) $$\sigma =\frac{ds}{dt}=-divI_{s}+\sigma_{i}=\sigma_{e}+\sigma_{i}\;,$$

where $I_{s}$ is the entropy flow density across the system boundaries; σ is the rate of change of the local entropy of the system; $\sigma_{i}$ – local entropy production; $\sigma_{e}$ – part of the rate of change of local entropy, due to the interaction with the external environment.

For our task, the expression $\sigma_{i}$ is well known:

(20) $$\sigma_{i}=-\frac{I_{Q}}{T^{2}}\frac{\partial T}{\partial x}=I_{Q}X^{\left(i\right)},$$

where $I_{Q}$ is the heat flow density and $X^{\left(i\right)}=-T^{-2}\partial T/\partial x$. It is obvious that equation (20) is a particular case of a more general expression:

(21) $$\sigma_{i}=\frac{ds_{i}}{dt}=\sum_{n}X_{n}^{\left(i\right)}I_{n}^{\left(i\right)},$$

where$X^{\left(i\right)}$ and $I^{\left(i\right)}$ – the generalized force and flow responsible for the production of entropy $\sigma_{i}$.

It is also well known that $I_{s}=I_{Q}/T$. Therefore (see (19)),

$$\sigma_{e}=-divI_{s}=-\frac{\partial }{\partial x}\left(\frac{I_{Q}}{T}\right)=-\frac{\partial I_{Q}}{\partial x}\frac{1}{T}+\frac{I_{Q}}{T^{2}}\frac{\partial T}{\partial x}\;.$$

Let the system be in a stationary state. Then ${\left(\partial I_{Q}/\partial x\right)}_{st}=0$ (“*st*” means “stationary value”). The latter follows from the thermal conductivity equation. Consequently, in a stationary state $\sigma_{e}=I_{Q}T^{-2}\partial T/\partial x$. We make the obvious notation: $X^{\left(e\right)}=T^{-2}\partial T/\partial x$. As a result, we get:

(22) $$\sigma_{e}=I_{Q}X^{\left(e\right)}\;.$$

If we take into account (18), then expression (22) admits a generalization similar to (21):

(23) $$\sigma_{e}=\frac{ds_{e}}{dt}=\sum_{n}X_{n}^{\left(e\right)}I_{n}^{\left(e\right)},$$

where $I^{\left(e\right)}$ and $X^{\left(e\right)}$ – the generalized flow and the generalized force responsible for the entropy change due to the interaction with the external environment.

It follows from (19), (20) and (22) that in the stationary state

(24) $$\sigma =\frac{ds}{dt}=\sigma_{e}+\sigma_{i}=I_{Q}X^{\left(e\right)}+I_{Q}X^{\left(i\right)}=I_{Q}(X^{\left(e\right)}+X^{\left(i\right)})=I_{Q}X^{\left(\sigma \right)}\;,$$

where $X^{\left(\sigma \right)}=X^{\left(e\right)}+X^{\left(i\right)}$.

Expression (24) admits a generalization similar to (21) and (23) (see also (17)):

(25) $$\sigma =\frac{ds}{dt}=\sum_{n}X_{n}^{\left(\sigma \right)}I_{n}^{\left(\sigma \right)},$$

where $I^{\left(\sigma \right)}$ and $X^{\left(\sigma \right)}$ respectively are the generalized flow and the generalized force responsible for the change in the entropy *S* of the system.

In the stationary state, the change in the entropy of the system over time is equal to zero:

(26) $$\sigma_{st}={\left(\frac{ds}{dt}\right)}_{st}=0\;.$$

Therefore, in a stationary state $\sigma_{e}+\sigma_{i}=0$. But $\sigma_{i}=I_{Q}X^{\left(i\right)}\neq 0$. From this we get the obvious ratios: $\sigma_{e}=-\sigma_{i}$ and $X^{\left(e\right)}=-X^{\left(i\right)}$ (the latter follows from (20) and (22)).

Thus, in order the entropy of the system does not change over time in a stationary state it is necessary to compensate the generalized force $X^{\left(i\right)}$ associated with the production of entropy $\sigma_{i}$ by the generalized force $X^{\left(e\right)}$ associated with interaction with the external environment. This means that existence of $X^{\left(e\right)}$ follows from physical considerations, but not just from mathematical ones. The last statement indicates the consistency of expression (24) and, as a consequence, of formula (25).

Let us note that in the general case $I^{\left(\sigma \right)}\neq I^{\left(i\right)}$. Indeed, even in the stationary state (the simplest case), the equality to zero of the generalized force $X^{\left(\sigma \right)}$ ($X^{\left(\sigma \right)}=X^{\left(e\right)}+X^{\left(i\right)}=-X^{\left(i\right)}+X^{\left(i\right)}=0$) means the absence of the generalized flow caused by this force: $I^{\left(\sigma \right)}=0$. This conclusion corresponds to the linear ratio between generalized flows and forces in Onsager’s theory:

(27) $$I^{\left(\sigma \right)}={\left(\frac{\partial I^{\left(\sigma \right)}}{\partial X^{\left(\sigma \right)}}\right)}_{eq}X^{\left(\sigma \right)}=L^{\left(\sigma \right)}X^{\left(\sigma \right)}=L^{\left(\sigma \right)}\cdot 0=0\;\left(\text{in}\mathrm{the}\;\mathrm{stationary}\;\mathrm{state}\right),$$

where $L^{\left(\sigma \right)}$ – kinetic coefficient.

It is obvious that (27) does not contradict (24). Indeed, in the stationary state is performed

(28) $$\sigma =I^{\left(\sigma \right)}X^{\left(\sigma \right)}=I_{Q}X^{\left(\sigma \right)}=0\;.$$

However, it does not follow from this expression that both flows are equal to each other. From (28) it follows only that the multiplications $I^{\left(\sigma \right)}X^{\left(\sigma \right)}$ and $I_{Q}X^{\left(\sigma \right)}$ are equal to zero. Concretely in this task, expression (28) corresponds to the following equality: $\underset{I^{\left(\sigma \right)}X^{\left(\sigma \right)}}{\underbrace{0\cdot 0}}=\underset{I_{Q}X^{\left(\sigma \right)}}{\underbrace{I_{Q}\cdot 0}}=0\;.$

So, in a stationary state $X^{\left(\sigma \right)}=0$ and $I^{\left(\sigma \right)}=0$. At the same time it is enough to change temperature difference between thermostats by a small predetermined amount in order the system leaves a stationary state (remaining in the field of the linear ratio of the generalized flows and forces). As a result the equality to zero is violated in (26) and

(29) $$\sigma_{e}\neq -\sigma_{i}\;\;\mathrm{and}\;\;X^{\left(\sigma \right)}\neq 0\;.\;$$

Then, according to (27) $I^{\left(\sigma \right)}\neq 0$. This flow seeks to bring the system to a new stationary state.

Let's remind that in the “*Generalized theorem on the minimum rate of change of entropy near the stationary state of the system*” section we considered exactly this situation on the example of Prigogine’s task. When the partition between vessels is removed the state of the system is nonstationary for some time. Consequently, ratios (29) are fulfilled. Therefore, a flow occurs (flows, if diffusion is taken into account), due to which the system goes into a stationary state with σ=0. The total entropy of the system changes during this transition and the sign of its change is determined from the conditions described in SCEC.

Let's notice that formula (25) becomes unpromising without SCEC. It is useless to apply it because of the uncertainty of the σ sign in the nonstationary state (this uncertainty follows from the uncertainty of the $\sigma_{e}$ sign, which in turn follows from the uncertainty of the $d_{e}S$ sign – see (15)).

2. Another important question relates to the values $L_{nk}$ designated by us as kinetic coefficients. But whether they are those in our work? Some of our critics doubt it. As an argument, they assert that these values do not satisfy the linear ratio between generalized flows and forces in our approach. The reason for this is that, in their opinion, the linear ratio is possible only if the derivatives of the kinetic coefficients with respect to the thermodynamic forces are equal to zero: $\partial L_{nk}/\partial X_{k}=0$

This is a serious objection. With its help formula (5) comes under criticism primarily. This is due to the fact that its derivation requires $L_{22}$ coefficient to be a constant value with respect to $X_{2}$, i.e. that $\partial L_{22}/\partial X_{2}=0\;.$

In response, let us note that in our description of the Prigogine task (as in [1]) $L_{22}$ is the “direct” kinetic coefficient of the diffusion process. Since this process is not supported from the outside, then $\sigma_{e}^{d}=0$ for it and $\sigma_{i}^{d}$ only remains (the “*d*” index indicates the diffusion process). In other words, the conditions adopted in [1] for the diffusion process are also preserved in our approach. In particular, the condition of $L_{22}$ constancy is preserved, which eliminates the stated claim to formula (5) (but does not guarantee the constancy of other coefficients). Let's notice that in the Prigogine model the requirement of constancy of kinetic coefficients is one of the restrictions imposed on the application field of his theorem on the minimum entropy production.

We support this requirement and confirm the restriction imposed by it on the application field of the results obtained in our work.

However, we cannot agree that the $\partial L_{nk}/\partial X_{k}=0$ condition is obligatory for the process to be considered linear. Indeed, in accordance with the well-known provisions of nonequilibrium thermodynamics, the linear ratio between generalized flows and forces ($I_{n}=\sum_{}L_{nk}X_{k}$) follows from the fact that the generalized forces are small near equilibrium. Let's remind that due to this fact we can decompose generalized flows by generalized forces into a Taylor series and neglect members with a higher order of smallness than the first (see, for example, [4]):

$$I_{n}=\underset{=0}{\underset{︸}{I_{eq}}}+\sum_{k}{\left(\frac{\partial I_{n}}{\partial X_{k}}\right)}_{eq}X_{k}+\frac{1}{2!}\sum_{k}{\left(\frac{\partial^{2}I_{n}}{\partial X_{k}^{2}}\right)}_{eq}{\left(X_{k}\right)}^{2}+\ldots +\frac{1}{n!}\sum_{k}{\left(\frac{\partial^{n}I_{n}}{\partial X_{k}^{n}}\right)}_{eq}{\left(X_{k}\right)}^{n}+\ldots$$

where “*eq*” means “equilibrium value”.

The kinetic coefficient is entered as $L_{nk}={\left(\partial I_{n}/\partial X_{k}\right)}_{eq}$. Therefore

(30) $$I_{n}=\sum_{k}L_{nk}X_{k}+\frac{1}{2!}\sum_{k}{\left(\frac{\partial L_{nk}}{\partial X_{k}}\right)}_{eq}{\left(X_{k}\right)}^{2}+\ldots +\frac{1}{n!}\sum_{k}{\left(\frac{\partial^{n-1}L_{nk}}{\partial X_{k}^{n-1}}\right)}_{eq}{\left(X_{k}\right)}^{n}+\ldots \approx \sum_{k}L_{nk}X_{k}.$$

The second term in (30) is neglected because of ${\left(X_{k}\right)}^{2}$ smallness in comparison with $X_{k}$, but not because of the zero derivatives $\partial L_{nk}/\partial X_{k}$. However, according to some of our critics, $I_{n}=\sum_{}L_{nk}X_{k}$ only if $\partial L_{nk}/\partial X_{k}=0$. In our opinion, this tough statement is ambiguous.

Using a simple example, we will demonstrate two different results of the $\partial L_{nk}/\partial X_{k}$ calculation, depending on the method of choosing a value for the “role” of kinetic coefficient.

Let us consider a homogeneous system with a constant temperature gradient in the *x* direction.

Variant 1. The Fourier law: $I_{Q}=-\lambda \partial T/\partial x$. Assuming $X_{Q}=-\lambda \partial T/\partial x$ (in general form: $X_{Q}=-gradT$), we get: $L_{Q}=\lambda =const$. Hence $\partial L_{Q}/\partial X_{Q}=0$ .

Variant 2. Let's use known expressions taken, for example, from [1]: $X_{Q}=\partial T^{-1}/\partial x$ and $L_{Q}=\lambda T^{2}$. Let the constant temperature gradient be equal to δ: ∂T/∂x=δ=const. Then $X_{Q}=-T^{-2}\delta$ . Easy to make sure that $\partial L_{Q}/\partial X_{Q}=2\lambda T/2T^{-3}\delta =\lambda T^{4}/\delta \neq 0$ .

## Conﬂict of interest

The authors declare that there are no conﬂicts of interest.
